# Innovative nanoparticle-based therapeutic strategies against glioblastoma multiform: a focus on enhanced delivery systems and efficacy

**DOI:** 10.3389/fbioe.2025.1601673

**Published:** 2025-07-23

**Authors:** Kehan Wang

**Affiliations:** Hubei University of Chinese Medicine, Wuhan, Hubei, China

**Keywords:** glioblastoma multiforme, nano compounds, delivery systems, blood-brain barriers (BBB), blood-tumor barrier

## Abstract

Glioblastoma multiforme (GBM) is an exceedingly aggressive primary brain neoplasm characterized by a dismal prognosis owing to its invasiveness, heterogeneity, and immunity to conventional therapies. Conventional therapies, including surgery, chemotherapy, and radiotherapy, encounter constraints due to tumor evasion and physiological obstacles, such as the blood-tumor (BTB) and blood-brain barriers (BBB), which impact the treatment of GBM. Nanotechnology is employed to augment the permeability of anticancer agents through these barriers, thereby improving treatment efficacy and minimizing toxicity. Lipid-based nanoparticles, such as nanostructured lipid carriers (NLCs) and solid lipid nanoparticles (SLNs), offer drug encapsulation, stability, and controlled release, whereas metal nanoparticles, including gold and silver, augment imaging and photothermal therapy efficacy. This review investigates the traversal of nano carriers across the BBB and BTB, emphasizing the significance of dimensions, charge, and surface functionality, while underscoring the potential of nanotechnology in managing GBM. Advancements in nanomedicine possess the capacity to create more efficacious therapeutic strategies, markedly improving patient outcomes in the management of GBM.

## 1 Introduction

The National Brain Tumor Society identifies 120 distinct types of brain tumors, with astrocytoma, a tumor of astrocytes, being the most prevalent. The World Health Organization (WHO) classifies astrocytes into four grades, from benign to malignant, according to cell atypia, mitotic activity, and angiogenesis. World Health Organization GBM, a grade 4 astrocytoma, is an aggressive and infiltrative brain tumor characterized by an unfavorable prognosis and diminished survival rate, originating from lower-grade glial cells (Torp et al., 2022; Sereika et al., 2018; Wesseling and Capper, 2018). The survival rates for astrocytoma, encompassing GBM, are 40% in the first year, 17% in the second year, and less than 10% by the fifth year, with a higher prevalence in males (Claus et al., 2015). Glioblastoma multiforme is categorized into three types: IDH mutant glioblastoma, IDH wild-type glioblastoma, and GBM NOS, with symptoms varying based on the tumor’s location in the cerebral hemispheres (Stoyanov et al., 2022). Typical manifestations of the condition encompass cephalalgia, emesis, visual disturbances, convulsions, dysphasia, personality alterations, and deviations in standard physiological functions. The risk of GBM is affected by genetic syndromes such as Lynch syndrome, and Li-Fraumeni, in addition to environmental factors like radiation exposure. Present detection techniques encompass MRI spectroscopy, traditional MRI, and functional MRI (Orr et al., 2020; Ceglie et al., 2020).

**FIGURE 1:**
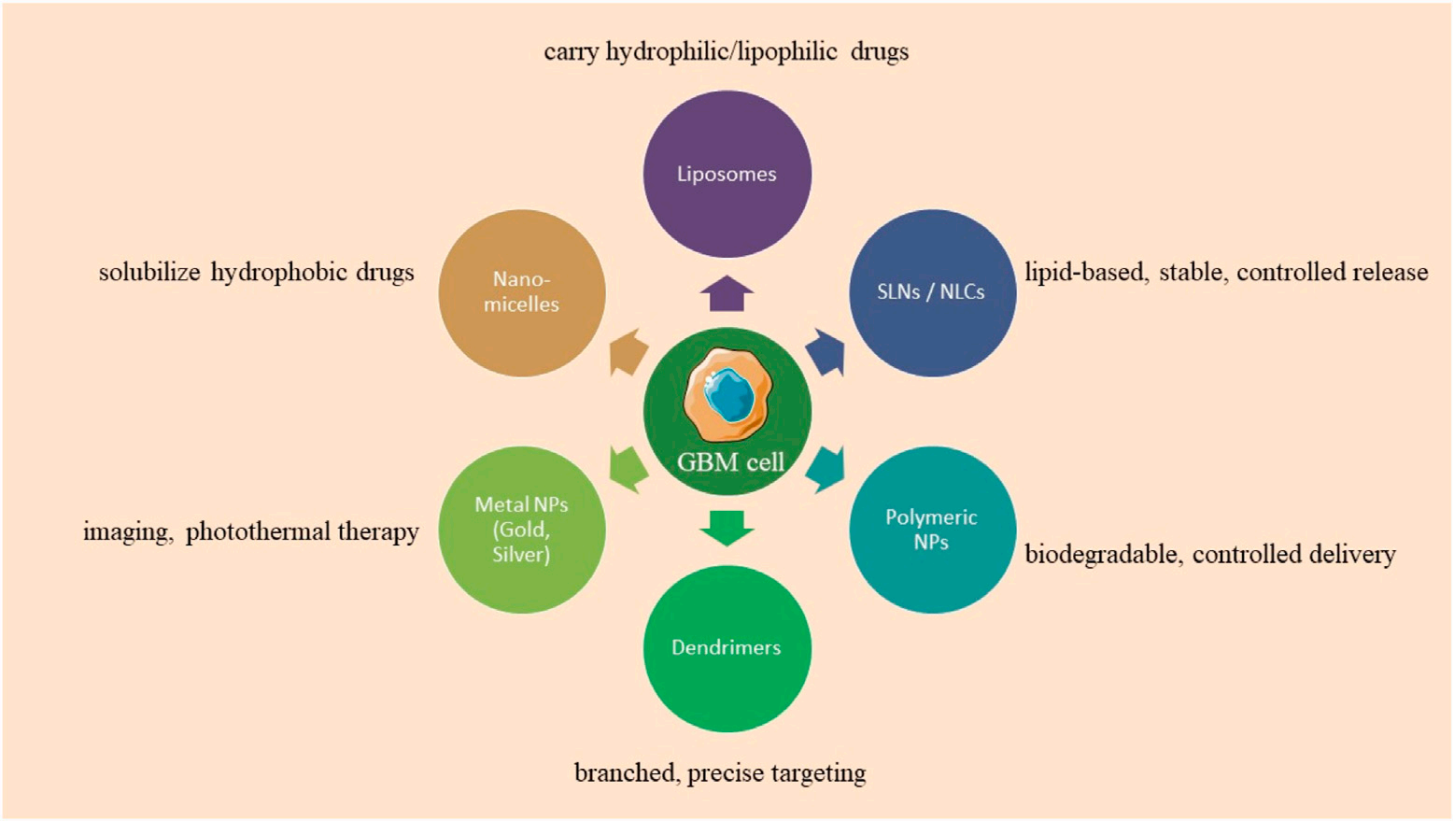
Types of nanoparticles used in glioblastoma treatment.

Numerous pharmaceuticals have been employed to treat and manage GBM; however, therapeutic efficacy is impeded by peripheral barriers such as the BBB and the blood-cerebrospinal fluid barrier (BCSFB). These barriers impede the entry of chemotherapeutic agents, resulting in the self-renewal of malignant cells and subsequent recurrence. First-line interventions, such as surgical excision, are impractical due to the distinctive attributes of GBM (Madadi and Sohn, 2024). Multiple strategies have been devised to surmount obstacles and limitations in the treatment of GBM, including modifying BBB permeability, employing ultrasound or radiation therapy to disrupt the BBB, and utilizing nanocarriers. Nanocarrier-mediated drug delivery is proficient in transporting pharmaceuticals to the central nervous system and tumor locations, as the nanoscale of the delivery systems facilitates access to the brain (as illustrated in Figure 1; Sethi et al., 2022). The method of administering nanoparticles influences drug bioavailability and internalization, which will be investigated subsequently in this review article.

## 2 Contemporary therapeutic methodologies and their constraints

Glioblastoma therapy is customized according to the neoplasm stage and the patient’s response to each intervention. Standard interventions for tumors encompass radiotherapy, chemotherapy, and surgical procedures aimed at debulking the tumor and diminishing its solid tissue mass (Fernandes et al., 2017). Surgery does not ensure the total elimination of tumor cells, requiring subsequent treatment with radiation or chemotherapy. Radiotherapy is a treatment modality that consists of 10–30 daily sessions, generally conducted 5 days per week, contingent upon the tumor type (Baskar et al., 2012). TMZ, sanctioned by the FDA for the management of glioblastoma multiforme, is administered daily to recently diagnosed patients at a dosage of 75 mg per square meter in conjunction with radiation therapy. Lomustine and bevacizumab are frequently utilized for progressive tumors, while the FDA has sanctioned tumor-treating field therapy (TTF) for both recurrent and newly diagnosed GBM (Cohen et al., 2005; Jezierzański et al., 2024). Conventional therapies exhibit adverse effects such as hemorrhaging, thrombus formation, tissue injury, increased infection risk, and protracted recovery, thereby constraining their clinical applicability. Tumor dislocation may transpire during surgery, and complete tumor excision is unfeasible due to the absence of delineation. Chemotherapy and radiation therapy may induce side effects such as alopecia, immunosuppression, anorexia, nausea, and vomiting (Kaur et al., 2022). Chemotherapeutic agents may acquire resistance through the expression of methylguanine-DNA methyltransferase (MGMT), which enhances protein expression and inactivates TMZ, resulting in 50% of tumors being unresponsive. TMZ is administered in cycles, permitting certain tumor cells to evade drug efficacy and proliferate as GICs. GICs, characterized by superior DNA repair mechanisms, resilient DNA damage response systems, and elevated efflux pumps, perform a crucial function in temozolomide resistance in GBM, rendering them essential targets for enhancing GBM treatment efficacy (Yu et al., 2019).

The BBB, a selectively permeable membrane, safeguards the brain from detrimental substances while permitting vital nutrients; however, it limits the passage of therapeutic agents, complicating the treatment of central nervous system disorders such as GBM. Therapeutic challenges for GBM encompass the BBB, tumor localization, cellular resistance, brain reparative capacity, malignant cell dissemination, capillary network disruption, and cellular accumulation, alongside the adverse effects of conventional therapies (Niazi, 2023). Radiotherapy resistance in GBM treatment arises from microenvironmental interactions among signaling pathways that sustain inherently radioresistant GBM stem cells. Challenges encompass tumor heterogeneity, hypermutation, drug efflux mechanisms, DNA damage repair pathways, immune evasion, micro-RNA resistance, oncogene-activated, and alternative splicing pathways. The existing treatment for glioblastoma patients is inadequate, requiring the creation of an effective delivery system for the conveyance of pharmaceuticals and biomolecules across the BBB (Osuka and Van Meir, 2017).

## 3 Requirement for nanoscale drug delivery systems

The aggressive characteristics of GBM result in an increased likelihood of tumor recurrence post-surgery and chemotherapy resistance, encompassing four primary concerns: the presence of the BBB and BTB, the distribution of drugs within the brain, and the physical and chemical properties of the pharmaceuticals. Diseases can compromise the structure and function of the BBB, resulting in a compromised and permeable BBB in GBM, diminished cerebral perfusion, and reduced treatment efficacy. For example, in high-grade astrocytoma, the BBB is replaced by the BTB, resulting in the inhibition of drug diffusion (Wu et al., 2021). The heightened permeability of GBM cells has resulted in a diminished efficacy of anticancer drug transport due to reduced specificity. Certain drug molecules are inappropriate for diffusion across the BBB owing to their physicochemical characteristics and physical impediments, resulting in multi-drug resistance. Consequently, nanocarrier delivery systems are essential for administering anti-cancer drugs to malignant and dormant cells, minimizing systemic side effects while preserving healthy cells (Khatoon et al., 2021; Fong, 2015). Nanotechnology is essential in the treatment of GBM through facilitating the efficient delivery of drugs across the BBB, enhancing tissue penetration, release, toxicity, and treatment precision. Nanoparticles encapsulate chemotherapeutic agents, augmenting efficacy. For example, polyethylene glycol-coated paclitaxel-PLGA nanoparticles extend circulation time in the bloodstream (Dam et al., 2025). Nanocarriers, owing to their dimensions and morphology, can readily infiltrate cells and tissues, facilitating enhanced drug loading. Their distinctive attributes, including size, zeta potential, and encapsulation efficiency, profoundly influence their biological capacity, therapeutic efficacy, and safety (Wei et al., 2023). The efficacy of nano-delivery systems for cerebral administration is influenced by parameters including nanometric dimensions, surface charge, morphology, and the molecular recognition and interplay between nanoparticles and cerebral targets. NPs, measuring between 1 and 1,000 nm, demonstrate enhanced infiltration and retention in cerebral tissues, with carrier systems in the 100–300 nm range yielding optimal outcomes. They can be modified with diverse compounds such as folic acid, PEG, glutathione, mannose, proteins, antibodies, and nucleic acids for efficient targeting (Formica et al., 2022). The augmented therapeutic efficacy of nanoparticles is due to their ability to safeguard against drug degradation, leading to enhanced, half-life, permeability, solubility, drug release, and tumor accumulation. Enhancement of drug penetration through the BBB can be achieved utilizing Nanocarriers such as magnetic nanoparticles, polymeric nanoparticles, cyclodextrins, lipid Nanocapsules, dendrimers, gold nanoparticles, quantum dots, and carbon nanotubes (Prieložná et al., 2024).

**FIGURE 2:**
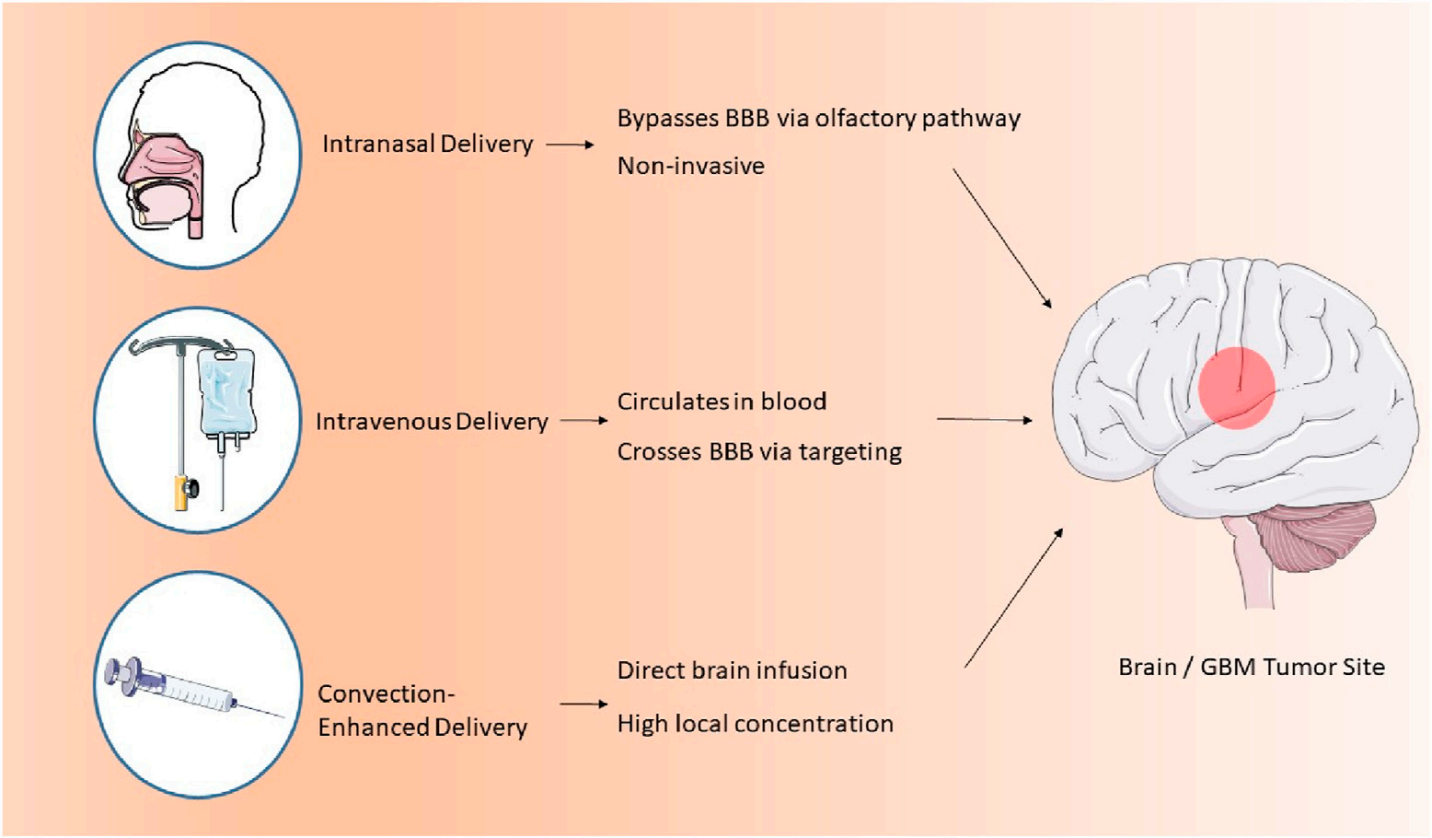
Three main routes of nanoparticle delivery to the brain in GBM therapy.

Convection-enhanced delivery (CED) is a method that facilitates the regulated infusion of anti-GBM pharmaceuticals into tumors via microcatheters. This method provides enhanced anti-tumor efficacy and reduced toxicity profiles relative to traditional dosage forms. A photopolymerizable hydrogel injection containing Temozolomide micelles was formulated, leading to reduced tumor weight in mice and enhanced drug release. This technique surpasses traditional dosage forms in efficacy. Direct systemic delivery is a minimally invasive technique for administering pharmaceutical compounds or nanostructured materials to the brain. This technique circumvents systemic circulation, resulting in reduced cerebral damage and improved outputs (D'Amico et al., 2021). A study involving rats with GBM indicated that direct systemic delivery significantly enhanced survival rates, thereby demonstrating its efficacy. Intranasal drug delivery is a favored approach for administering anti-GBM pharmacologically active compounds, providing noninvasiveness, elevated bioavailability, and improved patient adherence. Nonetheless, the absorption of nanoparticles through the nasal epithelium is impeded by inadequate transport and swift drug degradation. Permeation enhancers such as surfactants and phospholipids are utilized to augment diffusion capacity. Nanocarriers have demonstrated efficacy in regulating drug release and traversing the BBB, rendering them a potent instrument for the treatment of GBM. The dimensions of nanomaterials substantially influence cerebral targeting. Nanoparticles smaller than 20 nm are readily conveyed to the brain through extracellular transport and intracellular drug delivery via clathrin-dependent and caveolae-mediated endocytosis. Optimal nanoparticles must remain unaggregated and free from clumping. The properties of nanoformulations are determined by surface chemistry, particle size, charge, and the morphology of the nanocarrier (Qiu et al., 2025). Lipid NPs employed in anti-GBM therapeutics exhibit negligible mucociliary clearance and do not induce irritation or damage to the nasal mucosa (Mehta et al., 2023; Figure 2).

## 4 Trafficking of nanocarriers through the BBB

**FIGURE 3:**
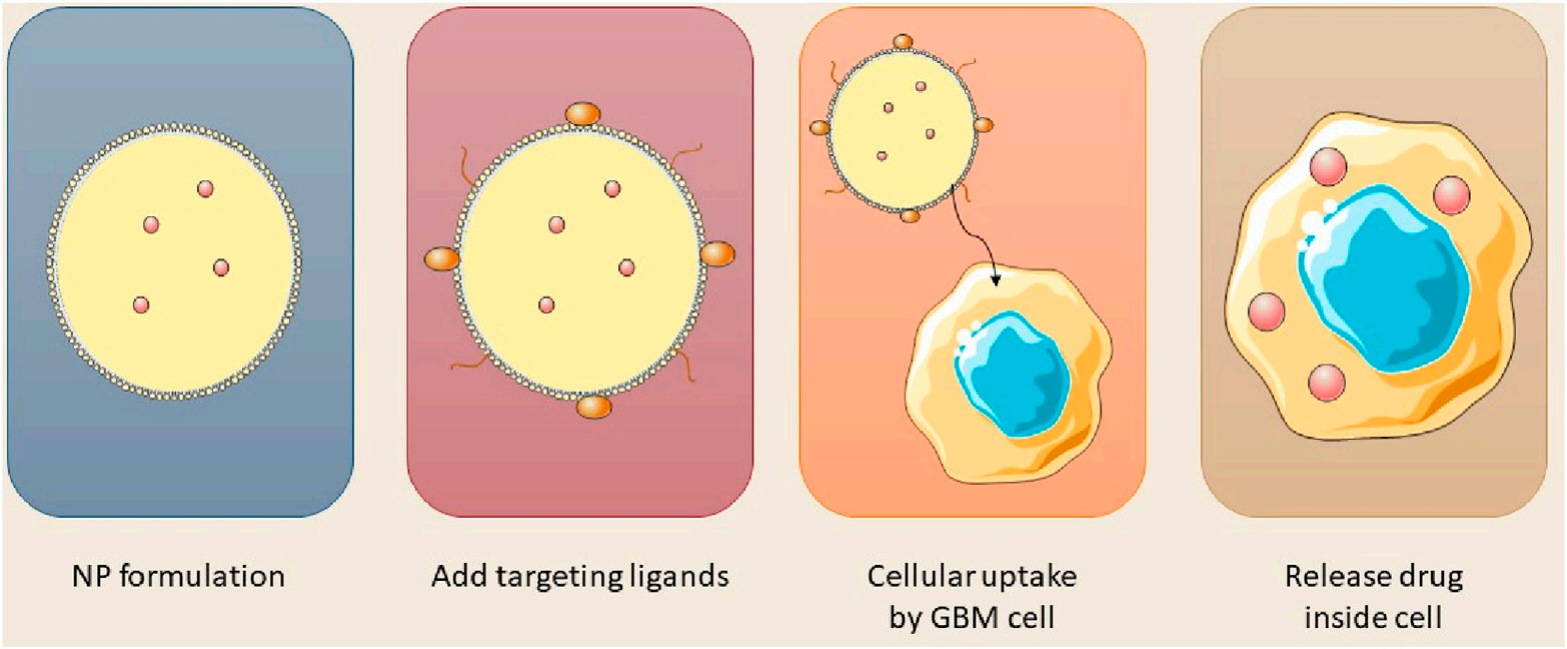
Stepwise design of nanoparticles for GBM cell entry and drug release.

The BBB is an essential membrane that governs cerebral fluid dynamics, sustains homeostasis, and safeguards against external agents. Therapeutic agents for neurological disorders can now be administered across the blood-brain barrier utilizing nanocarriers such as nanoparticles, micelles, and liposomes (Alahmari, 2021). The BBB is essential for safeguarding the brain, comprising multiple layers of physiological and enzymatic barriers that limit the passage of various chemicals. Microvascular endothelial cells (BMECs) govern the transport of vital substances between the circulatory system and the brain, maintaining equilibrium in the cerebral neurovasculature (Zhang et al., 2022). Mitochondria in capillary endothelium form tight junctions, preventing the entry of macromolecules. Transmembrane proteins preserve the integrity of cerebral blood vessels and enable the diffusion of small lipophilic molecules (Liang and Weber, 2014; Luissint et al., 2012). The brain’s basement membrane, consisting of Type IV collagen, laminin, pericytes, and fibronectin, functions as a conduit between the circulatory system and the brain; however, its restrictive characteristics impede the effective delivery of therapeutic agents. Numerous pharmaceuticals, particularly those targeting neurological disorders, encounter significant challenges in traversing the BBB owing to hydrophilicity, and elevated molecular weight and size (Tilling et al., 1998). Figure 3 illustrated the synthesis of nanoparticles for intracellular applications.

**FIGURE 4:**
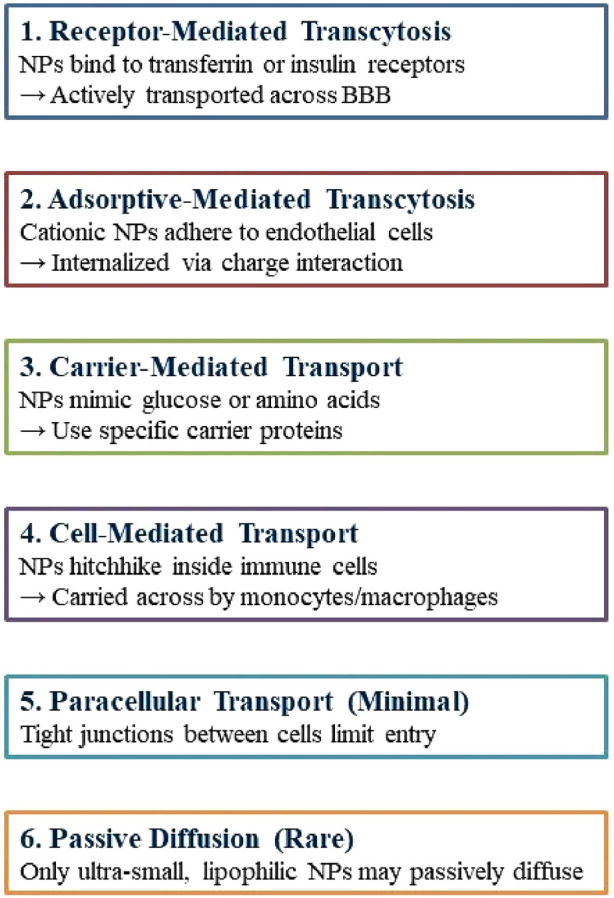
Mechanisms by which nanoparticles cross the blood–brain barrier.

Nanotechnology provides promising non-invasive treatments for GBM, minimizing physical trauma, and infection risk, and enhancing patient comfort. Engineered nanoparticles for optimal therapeutic delivery across the blood-brain barrier are exceptionally promising. Nanocarriers facilitate drug translocation across the BBB by optimizing size, surface functionalization and shape, thereby targeting endothelial cells with ligands (Tang et al., 2021; Akhter et al., 2021). Insulin, low-density lipoprotein receptor (LDLR), and transferrin are frequently utilized receptors in medicine. NPs can engage with BBB cell membranes via electrostatic interactions, facilitating their transport without the necessity for specific targeting ligands. The traversal of the BBB can occur via adsorptive-mediated transcytosis, cell-mediated transport, carrier-mediated transport, receptor-mediated transcytosis, paracellular transport, and passive transcellular diffusion (as depicted in Figure 4; Xiao and Gan, 2013; Zhang W. et al., 2021).

## 5 Nanocarriers for GBM therapy

Researchers are investigating nanoformulation in healthcare due to its size constraints, diminished self-aggregation, uniform size distribution, suitable excretion without negative effects, and gradual cargo release. The BBB, a selectively permeable membrane, enlarges with tumor growth, facilitating nanoparticle endocytosis, while diverse nanoformulations such as polymeric, lipidic, dendrimeric, carbonaceous, magnetic, and metallic act as effective drug delivery systems (Joseph et al., 2023).

### 5.1 Lipid nanoparticles

**FIGURE 5:**
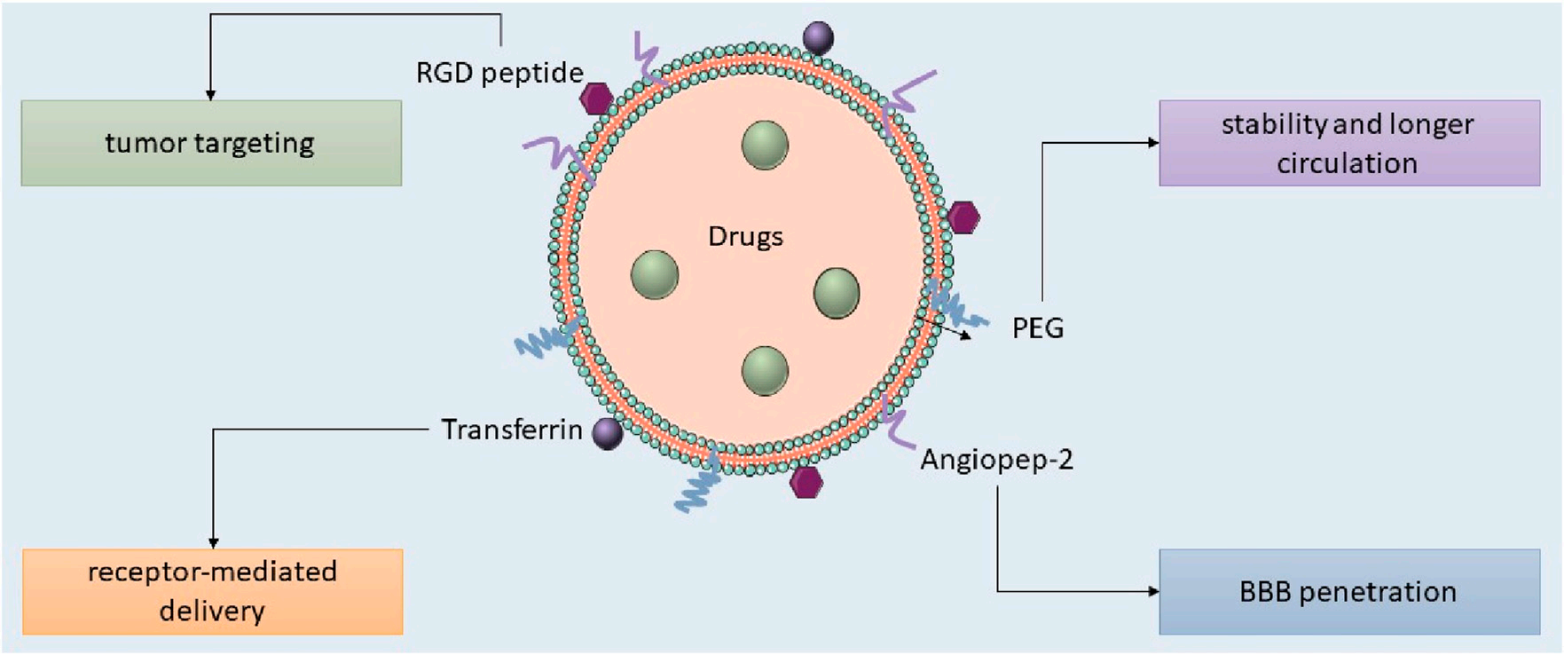
Targeted lipid nanoparticles for GBM therapy.

Lipid nanocarriers, including liposomes, SLNs, and NLCs, are favored in cancer research for their biocompatibility, biodegradability, controlled release, drug entrapment, and scalability, with SLNs frequently employed for the administration of pharmaceuticals to the central nervous system. Kim et al. developed an immunoliposome nanocomplex adorned with an antitransferrin receptor antibody, facilitating its effective passage across the blood-brain barrier, consequently diminishing MGMT expression in TMZ-resistant GBM cells and enhancing their susceptibility to TMZ (Kim et al., 2014). Lipid nanoparticles can be engineered for active targeting, enhancing their circulation longevity, augmenting cellular uptake and therapeutic efficacy, and reducing systemic toxicity. Lipid nanocarriers for GBM treatment can be engineered with ligands to disrupt endogenous transport mechanisms (as illustrated in Figure 5; Tang et al., 2023; Elmowafy and Al-Sanea, 2021). Lipid-based magnetic nanovectors, loaded with TMZ, synergistically combine magnetic hyperthermia and chemotherapy, resulting in TMZ sensitization, enhanced apoptosis, and inhibition of tumor cell migration (Beola et al., 2023).

#### 5.1.1 Liposome nanoparticles

Liposome NPs are spherical lipid vesicles containing a central aqueous core surrounded by phospholipid bilayers, with lamellarity determined by the number and size of the bilayers. They function as efficient drug carriers due to their ability to transport both hydrophilic and lipophilic molecules, while also being biocompatible, non-immunogenic, non-toxic, and biodegradable. The clinical applicability of liposomes is constrained by elevated production costs, drug expulsion and leakage, and a brief lifespan. Improvement can be attained by altering their surface and extending drug release (De Leo et al., 2022). The research created DOX-encapsulated angiopep-2 modified liposomes (AL) for the treatment of GBM by targeting the overexpressed lipoprotein receptor proteins in GBM cells (Danyu et al., 2011). Liposome-encapsulated DOX was administered to glioma-bearing mice on days 2, 5, and 8, leading to the most significant reduction in tumor size. Liposomes effectively administered DOX, diminishing GBM cell proliferation and inducing tumor cell mortality. DOX and liposomes demonstrated moderate toxicity, resulting in body weight increases of 0.86% and 6.43%, respectively, during the treatment period. DOX and liposomes exhibited moderate toxicity, resulting in body weight increases of 0.86% and 6.43%, respectively, during the treatment period. Nordlin et al. (2017) formulated TMZ liposomes to augment the effectiveness of drug by concentrating TMZ liposomes at tumor locations, leading to increased survival rates in rats with GBM. They discovered that liposomes-TMZ therapy significantly reduced GBM cell proliferation and prolonged survival compared to both free TMZ and free liposomes. Research on GBM in rodent models revealed that liposomes-TMZ led to extended survival compared to free TMZ or free liposomes, that it presented their potential for anticancer drug delivery across the BBB (Nordlin et al., 2017; Amarandi et al., 2022).

NPs engage with plasma proteins, forming a protein corona (PC) that interacts with cellular and biological barriers. Apolipoprotein E (ApoE) facilitates lipid transport between the brain and plasma, enabling drug delivery via LDL receptors on cancer cells. The Aβ-CN peptide served as a ligand to formulate ApoE-rich PCs with brain-targeting capabilities, essential for penetrating the BBB and administering drugs to glioma cells (Zhang ZA. et al., 2021).

#### 5.1.2 SLNs and NLCs

SLNs are colloidal pharmaceutical delivery systems composed of solid lipids, ranging from 50 to 1,000 nm in diameter, characterized by high stability, biocompatibility, low cost, and low toxicity, and include surfactants, co-surfactants, and water. SLNs enhance formulation stability by safeguarding the drug within the matrix; however, their loading capacity is constrained due to their optimal lattice structures (Mishra et al., 2018; Queiroz and Muehlmann, 2024). Liquid lipids were employed to augment the flexibility of lipid particles. Second-generation lipid nanoparticles, referred to as NLCs, are composed of both solid and liquid lipids, resulting in an imperfect crystalline structure that enhances drug retention and minimizes loss during storage (Mall et al., 2024). Qu et al. evaluated SLNs, TMZ-loaded, NLCs, and PNPs for GBM treatment, concluding that TMZ-SLN displayed the highest drug release rate, whereas TMZ-PNP exhibited the lowest release rate (Qu et al., 2016). TMZ-NLCs exhibited enhanced cytotoxicity against U87 cells, with IC50 values 4–7 times greater than those of TMZ-SLNs and TMZ-PNPs, indicating their capacity to diminish malignant glioma cell viability, and showed *in vivo* antitumor efficacy in murine models. The research indicated that TMZ-NLCs exhibited the most significant tumor regression in animals, achieving rates of tumor growth inhibition of 85%, 59%, 45%, and 27%, implying that NLCs may be efficacious for the management of malignant glioblastoma multiforme (Song et al., 2016). Kadari and others created docetaxel-loaded SLNs that were modified with angiopep-2 and tested how well they worked in C57BL/6 mice that had GL261 mouse glioma cells, using a dose of 10 mg/kg for 3 weeks. SLNs improved the drug’s half-life and facilitated the accumulation of encapsulated docetaxel in glioma, yielding a mean survival time of 39 days for subjects, in contrast to 24 days for unencapsulated docetaxel. The mice administered SLN displayed no clinical toxicity and negligible fluctuations in body weight (Kadari et al., 2018). The study by Wang et al. demonstrated that SLNs incorporating CAT3 enhanced the conversion of a novel prodrug for TMZ-resistant glioblastoma multiforme in rats, thereby diminishing drug resistance (Wang H. et al., 2021). TPGS-SLNs enhance passive cerebral targeting of trans-resveratrol (RSV) in glioma therapy, increasing brain accumulation by 9.23-fold relative to RSV solution, and demonstrating efficacy in rats (Gorain et al., 2018). Song et al. (2016) formulated TMZ NLCs for the management of GBM, incorporating the arginine-glycine-aspartic acid (RGD) peptide for targeted delivery across the BBB. The RGD peptide efficiently associates with GBM cells and tumor vascular endothelial cells, as evidenced in an orthotopic subcutaneous animal model utilizing BALB/c nude miceRGD-TMZ NLCs exhibited a reduction in tumor volume exceeding 80%, outperforming standard TMZ by a factor of three. H7K(R2)2, Folic acid, and cRGDfK, were employed to target glioblastoma cells within their acidic tumor microenvironment. The combination of atorvastatin and curcumin with nanostructured lipid carriers markedly diminished tumor size in an orthotopic xenograft model following a single injection. Researchers created positively charged chitosan-coated nasal lavage carriers (NLCs) to improve intranasal cerebral drug delivery, minimize dosing frequency, and enhance mucoadhesion within the nasal cavity (Gartziandia et al., 2015).

### 5.2 Polymer nanoparticles (PNPs)

Polymer nanoparticles are employed in the production of polymeric nanoparticles, which are submicron-sized carriers for anticancer therapeutics. Pharmaceuticals are delivered to the brain utilizing biodegradable and biocompatible polymers such as PGA, PLA, PBCA, PCL, and PLGA, which degrade into lactic and glycolic acids, eliminated through the Krebs cycle (Elmowafy et al., 2023). Kreuter et al. (1995) synthesized PBCA nanoparticles coated with p80 to enhance drug delivery across the blood-brain barrier into cerebral tissue, providing advantages including compatibility with pharmaceuticals, stability, optimized release kinetics, and drug protection. The platform was evaluated for the delivery of various anticancer agents in the treatment of GBM. Etoposide and Paclitaxel, both inhibitors of microtubule depolymerization, are extensively utilized in the treatment of various human malignancies, including GBM. Maliki et al. proposed the co-delivery of PTX and ETP using mPEG-PLGA nanoparticles because of their insufficient aqueous solubility and permeability through the blood-brain barrier. The NPs, synthesized via nanoprecipitation and coated with methoxy-PEG, measured under 150 nm and exhibited high encapsulation efficiency for ETP and PTX. The nanoparticles, measuring less than 150 nm, exhibited encapsulation efficiencies of 92.5% for PTX and 86.2% for ETP. The NPs exhibited prolonged drug release over 6 days, and a 216 nm PLGA nanoparticle containing 10% paclitaxel effectively traversed the blood-brain barrier to infiltrate brain tissues, addressing the obstacle of BBB penetration. Paclitaxel accumulates in the brain, liver, and kidneys, exhibiting a slower release when administered intranasally. PTX-PLGA nanoparticles exhibit anti-proliferative effects, particularly at elevated concentrations, in glioblastoma cell lines (Maleki et al., 2021).

Iguratimod, an anti-inflammatory compound, can sensitize TMZ-resistant GBM cell line U251 TMZ-R when encapsulated in PLGA, whereas PCL, a composite material, can improve sustained drug delivery in tumor peripheries (Younis et al., 2019). Acetylated dextran, a semisynthetic polymeric nanoparticle, emulates the membrane of GBM cancer cells, demonstrating a release rate surpassing 60% at acidic pH within 24 h (Lara-Velazquez et al., 2020).

FDA-approved bevacizumab is utilized for the treatment of progressive GBM in patients unresponsive to TMZ; however, its failure to traverse the blood-brain barrier may result in off-target effects. Sousa et al. formulated bevacizumab-encapsulated PLGA nanoparticles for olfactory delivery to the brain, improving drug retention, patient adherence, and reducing dosing frequency. The altered solvent emulsification-evaporation method produced a PDI of 0.056 and an encapsulation efficiency of 82.47%. The formulation’s regulated release of bevacizumab PLGA nanoparticles enhanced cerebral delivery by 14% after 7 h, resulting in a 7-day augmentation of cerebral exposure and heightened concentration (Sousa et al., 2019). The study revealed that bevacizumab was exclusively present in the brain of the bevacizumab-loaded PLGA NP group after 14 days of treatment, whereas free bevacizumab was detected in the liver and lungs, but absent in the brain. A study integrates nonviral DNA with biodegradable poly (-amino ester) nanoparticles to address GBM, specifically focusing on brain tumor-initiating cells (BTICs), which are accountable for tumor development and resistance to treatment. DNA encoding DsRed was effectively transfected into BTIC sample JHGBM-551 oncospheres using PBAE nanoparticles, achieving a transfection efficiency of 60% and up to 76% with 90% viability. These NPs demonstrated specificity for human BTIC and tumor cells in orthotopic tumors in nude athymic mice. *In vitro* evaluation of effectiveness demonstrated that nanoparticles could be preserved at −20°C for a minimum of 2 years (Sousa et al., 2017).

Photothermal therapy, a minimally invasive surgical approach, efficiently targets tumor cells with near-infrared light, safeguarding healthy tissues and minimizing recovery durations and complications (Jiang et al., 2021). Li et al. synthesized polymeric nanoparticles utilizing conjugated polymer (PBDPP) to efficiently deliver DPP to glioma cells via 3-phosphoethanolamine-N-[methoxy(polyethylene glycol) 2000 (DSPE-PEG). The synthesized PBDPP nanoparticles exhibited a photothermal conversion efficiency of up to 60% and absorption at 808 nm. They demonstrated photothermal heating of 62°C under laser irradiation, exhibiting considerable cytotoxicity against human U-87 MG glioma cells. The research indicated that, even in the absence of light, PBDPP nanoparticles eradicated 60% of glioblastoma cells, with cell mortality escalating to 95% when combined with laser irradiation at a low concentration. It was also indicated that PBDPP nanoparticles, upon irradiation, effectively ablate glioblastoma cells with superior spatiotemporal accuracy. PBDPP nanoparticles achieved complete tumor eradication in mice at a low dosage of 0.35 mg/mL under near-infrared irradiation, highlighting their potential as a low-dose, high-efficacy theranostic platform (Li et al., 2019). Bernal et al. conducted a study employing polymeric nanoparticles (PMNPs) for the delivery of TMZ in rats, illustrating their capability in drug monitoring through MRI. MRI scans revealed a substantial augmentation in PMNP distribution within the brain parenchyma following CED compared to bolus injection. Endocytosis served as the principal mechanism for PMNP absorption. Nonetheless, inadequate drug delivery indicated the necessity for a targeted strategy employing ligands (Bernal et al., 2014; Faraji et al., 2020).

#### 5.2.1 Nano micelles

Nano micelles are nanoscale drug delivery systems formed by the self-assembly of amphiphilic block copolymers in an aqueous environment, comprising two or three segments. Polymers self-assemble to form a hydrophobic core segregated from the aqueous environment by hydrophilic segments. Nano polymers such as polyethers polyesters, poly(L-lysine), poly(ε-caprolactone), poly (glycolic acid), and poly (α, β-aspartic acid), are suitable for hydrophobic segments. Nano micelles are capable of encapsulating chemotherapeutic agents, enhancing penetration of the blood-brain barrier, and enabling targeted delivery to tumor cells (Bose et al., 2021). Curcumin, a prime candidate for glioblastoma therapy, demonstrated substantial clinical advantages when encapsulated in PEG-coated PLA micelles. Curcumin-MPEG-PLA effectively inhibited the proliferation of GL261 cells and cancer cells, induced apoptosis, and suppressed tumor growth in murine models by inhibiting angiogenesis (Nowacka et al., 2025). Gao et al. have formulated nano-encapsulated rutin within micellar nanoparticles to enhance its antitumor efficacy, as its low hydrophilicity constrains its therapeutic potential. Rut-micelles, infused with rutin, exhibited considerable cytotoxicity and antimetastatic properties, indicating their potential in the treatment of GBM, as evidenced by their hemocompatibility and antimetastatic efficacy in a cell monolayer wound scratch assay (Gao et al., 2023). Doxorubicin, an anthracycline agent utilized for the treatment of solid tumors, was co-loaded with honokiol (HK), a bioactive compound derived from the bark of Magnolia officinalis, onto biodegradable micelles to enhance its bioavailability. The 34 nm micelles elicited a dose-dependent decrease in glioma cell viability, with DOX-HK-MPEG-PCL nanoparticles promoting enhanced apoptosis than DOX-MPEG-PCL and HK-MPEG-PCL.Both pharmaceuticals demonstrated high drug-loading efficiencies. Studies indicate that self-assembling nano micelles significantly impede glioma growth by enhancing apoptosis, diminishing cancerous growth, and hindering angiogenesis, in both *in vitro* and *in vivo* environments (Gao et al., 2017).

#### 5.2.2 Dendrimers

Dendrimers are nanoscale, radially symmetrical molecules comprising a central core, an intermediate shell, and an external shell. They are increasingly utilized in drug delivery and imaging because of their capacity to encapsulate or conjugate high molecular-weight compounds. Dendrimers, predominantly synthesized from polyamidoamine (PAMAM), are composed of diverse polymers including poly-L-lysine, poly (ether hydroxylamine), melamine, PAMAM, poly (propylene imine) (PPI), polyglycerol and poly (ester amine) (Abbasi et al., 2014; Bacha et al., 2023). Dendrimer-mediated therapy for glioblastoma multiforme is limited by insufficient cellular infiltration and drug conservation; however, Tween 80 and PPI dendrimers were formulated by Gajb et al. (2009) for the delivery of docetaxel to the brain for GBM treatment. Docetaxel dendrimers exhibited superior cytotoxicity compared to free drugs on U87 cells and markedly diminished tumor sizes *in vivo* in male albino rats with GBM. DTX-P80-PPI markedly enhanced the MST in rats with brain tumors compared to DTX-PPI or free DTX, underscoring the necessity for novel drug delivery systems. Tumor-associated macrophages (TAMs) represent viable targets for advancing effective cancer therapies owing to their function in modulating the immunological response to neoplasms (Duwa et al., 2019). The study by Sharma et al. indicates that glucose level modifications can markedly enhance tumor-associated macrophages and microglia targeting by improving brain penetration and cellular internalization (Sharma et al., 2021).

### 5.3 Metalic nanoparticles

Recent investigations into metallic NPs, including quantum dots, iron oxide, gold, silver, superparamagnetic iron oxide, and iron-free magnetic nanoparticles, show promise for cancer treatment (Sidhic et al., 2025). Metal-based nanoparticles proficiently transport agents such as DNA, RNA, and pharmaceuticals to tumor locations, inhibiting GBM proliferation, radioresistance, and tumor recurrence by traversing the BBB. Metal-based NPs utilize gold, silver, and various other metals, whereas magnetic nanoparticles concentrate on iron-based compositions. Yu et al. (2022) formulated TMZ encapsulated with targeted gold nanoparticles, which convert light into thermal energy, thereby initiating plasmonic photothermal therapy (PPTT). Superparamagnetic iron oxide nanoparticles (SPIONs) are magnetic inorganic nanoparticles that modulate their magnetization at designated temperatures. Magnetite and maghemite are essential for target specificity, hyperthermia, and MRI imaging. A study utilized magnetoelectric nanoparticles with a CoFe2O4@BaTiO3 core-shell composition to deliver MIA690, a growth hormone antagonist, to the U87-MG glioblastoma cell line (Israel et al., 2020; Stewart et al., 2018).

Gold nanoparticles (AuNPs) are under investigation for cancer therapy owing to their minimal toxicity, low immunogenicity, stability, biocompatibility, enhanced permeability, and innate immune activation characteristics (Gawel et al., 2022). Wang L. et al. (2021) engineered anti-EphA3-TMZ gold nanoparticles, which are TMZ-encapsulated gold nanoparticles functionalized with an antibody targeting the ephrin type-A receptor 3, for intranasal delivery in the treatment of GBM. The method focuses on active GBM cells without traversing the BBB, improving temozolomide and therapeutic efficacy while minimizing systemic toxicity and drug resistance; AuNPs are appropriate for intranasal administration. *In vitro* studies demonstrated that anti-EphA3-TMZ gold nanoparticles enhanced the apoptotic ratio and improved cellular uptake, exhibiting an IC50 that was 18.5 times lower than that of free drugs, thereby indicating heightened cytotoxicity in C6 glioma cells. This strategy diminished the upregulation of O6-methylguanine-DNA methyltransferase, which is responsible for resistance to TMZ. Functionalized AuNPs, including anti-EphA3-TMZ AuNPs, substantially diminished tumor size in orthotopic GBM-induced rats, prolonging median survival time and demonstrating their potential as an effective GBM delivery system.

Silver nanoparticles (AgNPs) represent a promising material for research in nanomedicine. Silver nanoparticles are in high demand owing to their distinctive physicochemical and biological characteristics, such as biocompatibility, elevated surface-to-volume ratio, strong antibacterial efficacy, remarkable surface plasmon resonance, ease of functionalization, and cytotoxicity towards cancer cells (Abbas et al., 2024). AgNPs modify membrane fluidity, facilitating entry and accumulation in cancer cells, resulting in cell death or diminished proliferative capacity, and potentially inducing premature apoptosis via altered signaling pathways (Takáč et al., 2023). Liu et al. assessed AgNPs in conjunction with a singular radiation dose for treating GBM utilizing c6 glioma cells implanted in rat brains and MRI to verify tumor proliferation. Silver nanoparticles (AgNPs), approximately 20 nm in diameter, were administered to rats 8 days’ post-tumor implantation, after which 10 Gy of radiation was applied. The research indicated that the groups receiving 10 and 20 g AgNPs in conjunction with radiation exhibited notable increases in life expectancy and cure rates at 200 days, with average survival durations of 100.5 and 98 days, respectively. The research indicated that radiation therapy and AgNPs effectively addressed cancer with minimal side effects, yielding average survival times of 24.5, 16.1, and 19.4 days, respectively (Liu et al., 2016).

**FIGURE 6:**
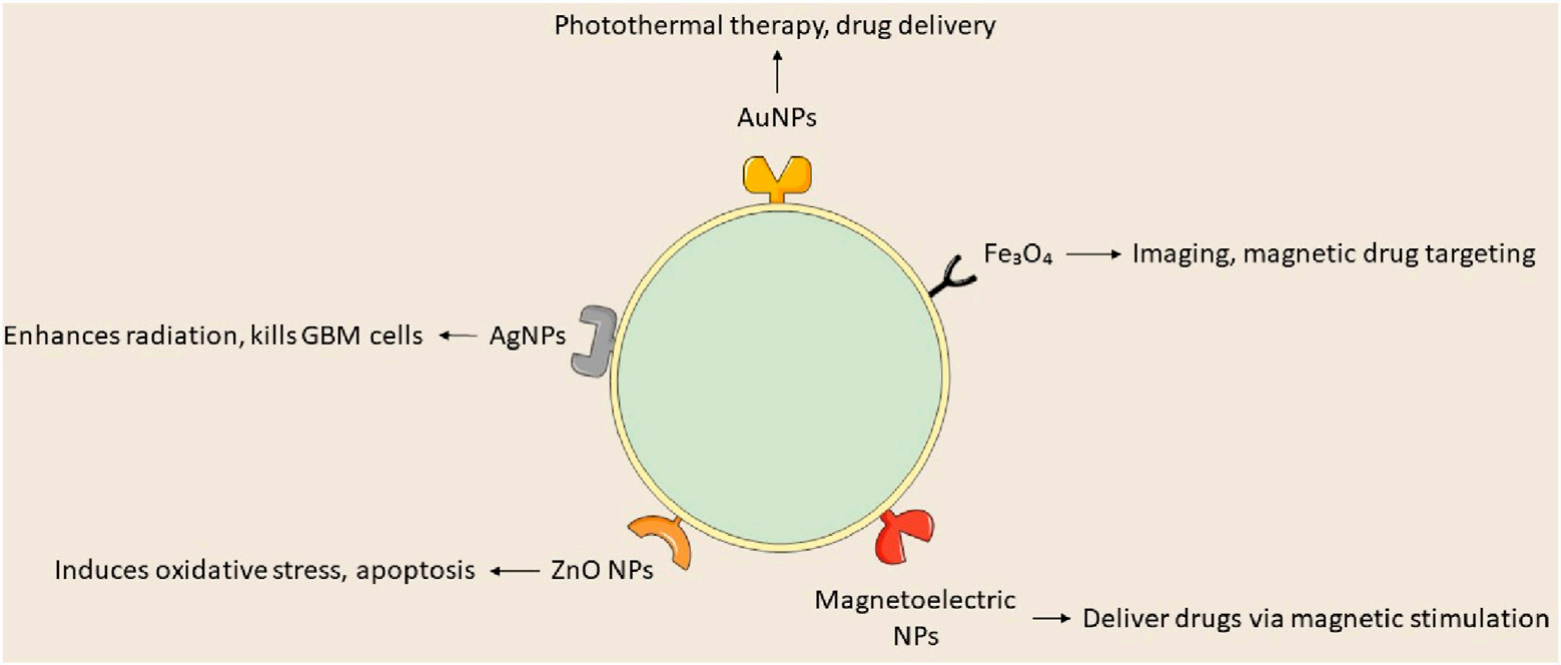
Types of metal nanoparticles and their roles in GBM treatment.

Iron oxide nanoparticles have demonstrated efficacy against multiple cancer types, including prostate, breast, liver, gastric, lung, ovarian, and colorectal cancers. Iron oxide nanoparticles are sanctioned for magnetic hyperthermia-assisted therapy of GBM, utilized as an adjunctive treatment alongside stereotactic radiotherapy for recurrent GBM. Nano-Cancer^®^ employs ferrous oxide particulates impregnated with amino silane to infiltrate tumor tissue, activated by an alternating magnetic field, resulting in thermal damage to GBM cells (García-Soriano et al., 2024; Manescu et al., 2024). Metal nanoparticles can be utilized clinically if hazards and toxicity are controlled throughout production and treatment; however, their diminutive size and extensive surface area enhance reactivity with biological targets. Products based on nanotechnology encounter safety and environmental concerns. Although metal nanoparticles are undergoing preclinical and clinical trials, their efficacy as cancer therapies is established. Nonetheless, further investigation is required to apply these findings in clinical environments (Khan et al., 2021; Nair et al., 2022; Figure 6; Table 1).

**TABLE 1:** Overview of diverse Nano compounds applied for GBM therapy.

Naocomponds	Main outcomes	References
Lipid Naocomponds		
TMZ-loaded CL–BC liposomes	TMZ-loaded cationic liposomes markedly improved antitumor effectiveness against the GBM U-87 MG cell line, especially in HUVECs, a recognized model of the BBB.	Arcella et al. (2018)
Doxorubicin liposomes	Doxorubicin liposomes markedly decreased tumor volume, allowing 57% of subjects to survive beyond 200 days after tumor implantation, attributed to increased cellular accumulation via IL-13 receptor expression.	Madhankumar et al. (2009)
Doxorubicin loaded SLNs	Permeability studies indicated that SLN enhanced the BBB permeability coefficient for DOX and suppressed the proliferation of U87 cells.	Kuo and Lee (2016)
Hyaluronan-decorated lipid nanoparticles (HA-LNP)	HA-LNP efficiently targeted GBM cells, resulting in substantial cytotoxicity in U87 GBM cells *in vitro* and delaying tumor proliferation in a subcutaneous tumor animal model.	Gibson et al. (2023)
Celecoxib (CLX) EGFR-targeted immunoliposomes	Encapsulating CLX demonstrated a 40% efficiency and prolonged drug release, with enhanced uptake in EGFR-overexpressing cells relative to nontargeted liposomes.	Limasale et al. (2015)
Sorafenib Lipid nanocapsules	SFN-LNCs exhibited enhanced cerebral targeting in U87MG-bearing mice, suppressing tumor cell proliferation and diminishing angiogenesis relative to control mice.	Clavreul et al. (2018)
Polymeric Nano compounds		
Curcumin dendrimers	Curcumin-PAMAM dendrimers markedly reduced the viability of all three glioblastoma cell lines: rat-F98, human-U87, and mouse-GL261.	Gallien et al. (2021)
mRNA-carbosilane Dendrimers	Dendrimers, specifically mRNA-dendrimers, have demonstrated the ability to alter surface marker expression in glioblastoma cell lines, glioblastoma stem-like cells, and induced pluripotent stem cells, displaying enhanced cytotoxicity towards tumor cells.	Knauer et al. (2023)
Anti-programmed death-ligand1 antibodies (aPD-L1) loaded into a redox-responsive micelles	The co-encapsulation technique effectively suppressed primary and recurrent GBM, increased cytotoxic T lymphocyte accumulation, and established lasting immunological memory in orthotopic GBM-bearing mice.	Zhang et al. (2024)
Poly (β-amino ester) s (PBAEs) nanoparticles for non-viral DNA delivery	PBAE-DNA nanoparticles exhibited a transfection efficiency exceeding 60% in orthotopic tumors induced in nude mice.	Guerrero-Cázares et al. (2014)
Metalated porphyrin-doped conjugated polymer nanoparticles (CPNs)	Cellular investigations demonstrated that CPNs displayed cytotoxic effects in MO59K and U-87 MG GBM cells, with enhanced cytotoxicity noted in T98G GBM cells.	Caverzán et al. (2020)
Lipid-polymer hybrid nanoparticles (LPHNs-cRGD)	LPHNs-cRGD suppressed MGMT expression, enhanced GBM cell vulnerability to TMZ, and extended the survival of tumor-bearing mice.	Yang et al. (2021)
Conjugated polymer nanoparticles (CPN) with metronomic photodynamic therapy (mPDT)	CPNs, upon exposure to mPDT, demonstrated preferential absorption by GBM cells, resulting in reduced tumor proliferation and enhanced cellular apoptosis in a GBM animal model.	Caverzán et al. (2023)
Diketopyrrolopyrrole (DPP)-based Conjugated Polymer NPs (CPNs)	Hyaluronic acid-conjugated CPNs demonstrated selective uptake in CD44-positive GBM patient-derived cells, affected by BBB permeability, concentration, and cell cycle phase.	Lubanska et al. (2022)
Borneol-modified docetaxel plus tetrandrine micelles	The application of borneol-modified micelles for the treatment of docetaxel and tetradrine enhanced bEnd.3 uptakes, facilitated drug penetration across the blood-brain barrier, suppressed cell proliferation, induced apoptosis, and reduced the expression of drug-resistant proteins.	Liu et al. (2024)
Doxorubicin-loaded PLGA nanoparticles	Modified PLGA nanoparticles conjugated with RGD ligand facilitate tumor-targeted delivery of doxorubicin, thereby improving tumor suppression in a C6 cell-implanted GBM animal model.	Chung et al. (2020)
TMZ and bortezomib (BTZ) PLGA nanoparticles	BTZ/TMZ nanoparticles significantly suppressed tumor cell viability and proliferation in GBM cells, while sparing healthy cells and demonstrating excellent biocompatibility.	Ramalho et al. (2023)
PAMAM dendrimer loaded with siLSINCT5 (NP- siRNA)	The dendrimer conjugated with siRNA effectively suppressed GBM by obstructing LSINCT5-activated signaling pathways and enhancing anti-tumor immunity through traversing the BBB.	Jin et al. (2021)
Metallic Nano compounds		
AuNPs (Gold nanoparticles)	Temozolomide-loaded gold nanoparticles demonstrated an 82.7% inhibition rate in cancer stem cells relative to free drugs, primarily attributable to diminished chemoresistance in patient-derived cancer stem cells.	Orza et al. (2013)
Temozolomide-coated nano zinc oxide particles	ZnO nanoparticles markedly induced apoptosis in U87 glioma cells, exhibiting an IC50 value of 30 g/mL.	Zheng et al. (2018)
Aptamer U2 conjugated AuNPs	U2-AuNP significantly suppressed the proliferation and invasion of U87 human glioblastoma cell lines, prolonging the survival of glioblastoma-bearing mice by obstructing the EGFR-related pathway.	Peng et al. (2020)
AgNPs (silver nanoparticles)	Chlorotoxin-conjugated PEGAgNPs exhibited considerable cytotoxicity in U87 cell lines, facilitating increased uptake into glioma cells owing to their targeting peptide.	Locatelli et al. (2012)
Au-CuO and CuO-ZnO nanoparticles	NPs interrupted the G2–M phase in C6 rat brain glioma and T98G human glioma cells, resulting in considerable cell mortality.	Dobrucka et al. (2019)
Zinc-doped copper oxide nanoparticles	Zn-CuO NPs suppressed cell proliferation, inhibited tumor growth, induced apoptosis, and produced reactive oxygen species, indicating potential applicability for patient’s resistant to TMZ.	Wu et al. (2018)

### 5.4 Polymer-lipid hybrid nanoparticles

**FIGURE 7:**
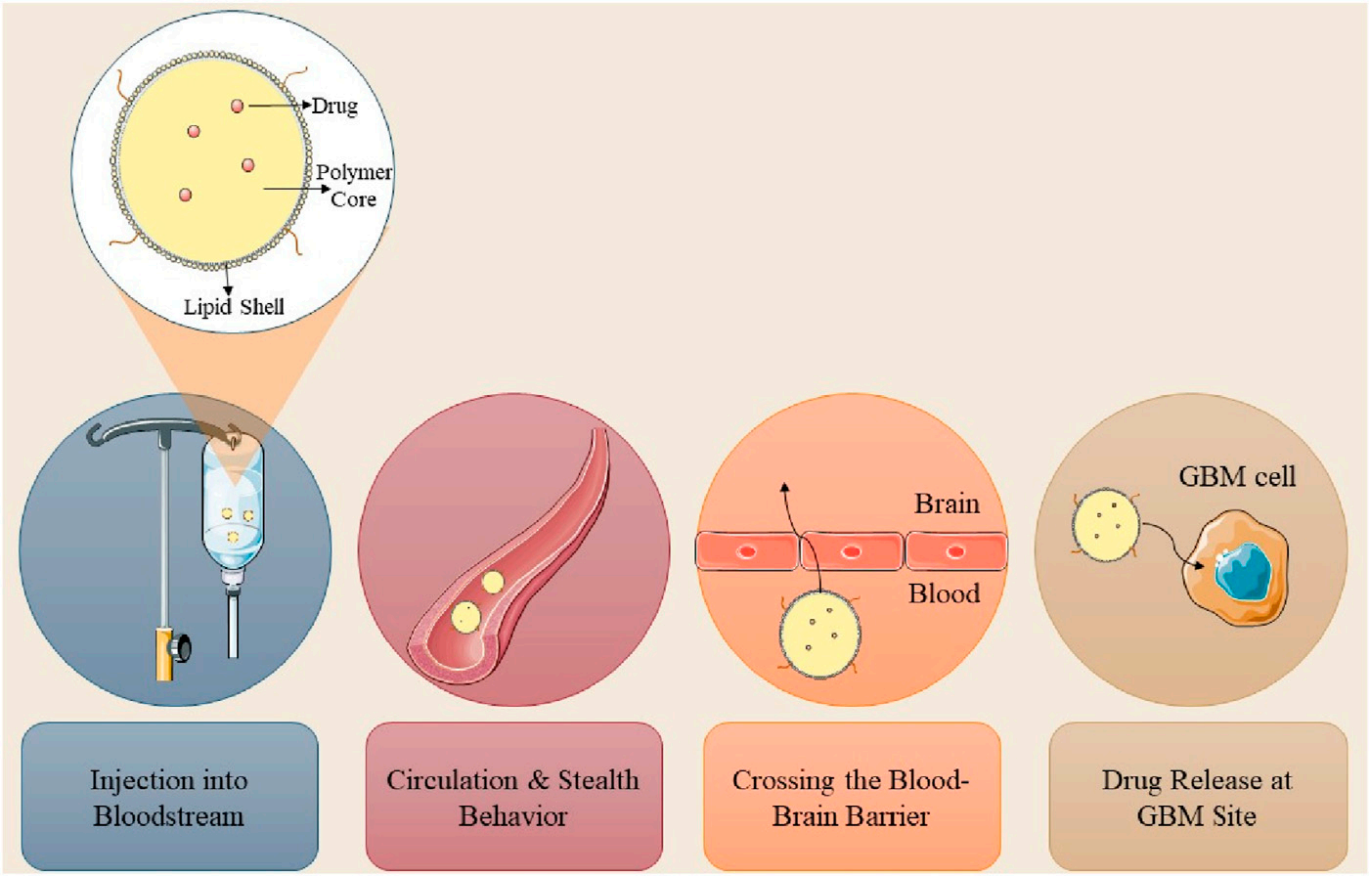
Mechanism of action of lipid–polymer hybrid nanoparticles in glioblastoma therapy.

Polymer lipid hybrid nanoparticles (PLHNPs) represent an innovative delivery system that integrates the characteristics of polymeric and lipid-based nanoparticles. The lipid core or shell is encased in a polymer matrix, ensuring structural integrity and safeguarding against premature degradation. This hybrid structure improves the encapsulation efficiency of phytochemicals and drugs, as well as their solubility, stability, and bioavailability. PLHNPs can mitigate challenges in phytochemical delivery by providing reduced particle size, elevated encapsulation efficiency, enhanced stability, and improved dissolution in gastrointestinal fluids. They also surmount constraints such as rapid metabolism and restricted bioavailability by encapsulating phytochemicals within hybrid matrices. PLHNPs can encapsulate and co-deliver two drugs with differing physicochemical properties, exhibiting synergistic therapeutic efficacy. Lipophilic medications are encapsulated within the polymeric core, whereas hydrophilic medications are confined within the lipid shell. PLHNPs serve as an effective nanocarrier for the oral chemotherapy of breast cancer. Comprising GRAS lipids and polymers, they provide diminutive particle size, substantial load capacity, stability, and solubility in gastrointestinal fluids. PLHNPs demonstrate enhanced intestinal absorption and bioavailability following ingestion. They possess the capability for precise delivery of chemotherapeutic agents, leading to enhanced therapeutic effectiveness and reduced dose-dependent toxicity. D-alpha-tocopheryl polyethylene glycol succinate (TPGS) serves as an emulsifier, solubilizer, and stabilizer in the development of nanocarriers. It improves drug solubility and stability, and may influence cancer cells. In a study, EXE-TPGS-PLHNPs were formulated to enhance therapeutic efficacy against MCF-breast cancer cells. The utilized polymer, lipid, and surfactant were polycaprolactone, phospholipon 90G, and a poloxamer-188 mixture. The findings indicated enhanced cellular uptake and cytotoxicity in MCF-7 cells. Moreover, The research assessed the *in vivo* anti-breast cancer effectiveness of PLHNPs in Balb/c mice bearing MCF-7 xenograft tumors, contrasting the outcomes with those of EXE-PLHNPs and free drug suspension (Rahat et al., 2024; Rizwanullah et al., 2020; Rizwanullah et al., 2023; Rizwanullah et al., 2021; Figure 7).

### 5.5 Other NPs

Researchers have created a nanosensor for identifying drug dosimetry patterns in intracranial tumors utilizing hydrogel-encapsulated carbon nanotubes. The sensor can effectively monitor spatiotemporal therapeutic data with minimal toxicity *in vivo*. Semiconductor nanoparticulate systems are developing as intelligent nanoparticulate systems characterized by their diminutive size, magnetic and optoelectronic properties, and facile excretion via renal filtration (Son et al., 2023). These NPs improve tumor imaging in both preclinical and clinical subjects by producing ROS within tumor cells. Nosrati and associates (Nosrati et al., 2022) engineered a nano radiosensitizer composed of bismuth sulfide and gold to improve CT scan imaging, resulting in substantial defects within Bi2S3 semiconductors for photothermal therapy in neoplastic cells. The combination, along with methotrexate and curcumin, effectively eliminated tumor growth in BALB/c mice following a single dose of irradiation.

Exosomes are generated within the endosomal system via the biogenesis of late endosomes. Intraluminal vesicles (ILVs) are generated when the membrane of the multivesicular body (MVB) invaginates. These ILVs encompass the intracellular contents and particular proteins, subsequently released into extracellular space upon fusion with the cell’s plasma membrane. Brain exosomes are essential for neuronal regeneration and development, and play a role in numerous neurological disorders. Extracellular vesicles (EVs) are emerging as a novel mechanism for material exchange within the nervous system, encompassing neuroactive substances. Exosomes participate in the synthesis of various substances by tumor cells, such as growth factors, metabolites, cytokines, and ions. They convey molecules such as histones, oncogenic entities, and tumor suppressors. Exosomes derived from GBMs can modify immune system function by affecting phagocytic capacity, altering protein surface characteristics, and influencing cytokine synthesis (Filannino et al., 2024). Recent research has emphasized the significance of exosomes in the lifecycle of invadopodia. Exosomes in mesenchymal cells can facilitate tumor proliferation and invasion by modifying the microenvironment. They adversely affect normal support cells and elicit atypical characteristics in GBM subtypes. Exosomes facilitate angiogenesis, an essential process in glioma progression, by delivering proteins and mRNA to vascular endothelial cells. Gliomas secrete factors such as EGFRvIII that promote angiogenesis. Exosomes play a vital role in regulating the proliferation and invasion of glioma cells, facilitating their survival and recurrence. They export pharmaceuticals from neoplastic cells, resulting in therapeutic resistance. Exosomes provoke fibroblastic responses, obstructing anticancer pharmaceuticals. They mediate tumor cell responses to hypoxia, influencing angiogenesis, tumor proliferation, and metastasis. Hypoxia influences the genetic and proteomic characteristics of tumor cells, while alterations in exosomal miRNA augment cellular signaling and motility, facilitating angiogenesis and promoting cellular proliferation (Luo et al., 2023).

Natural exosomes, capable of targeting specific cells, can traverse the blood-brain barrier under certain conditions. Exosomes from cancer cells can influence other cells via surface receptors, including heparan sulfate proteoglycans (HSPGs). Engineered exosomes can traverse the blood-brain barrier via receptor-mediated transcytosis. A study integrated methotrexate with engineered exosomes to target the low-density lipoprotein receptor in the blood-brain barrier, with the objective of developing a treatment for glioblastoma multiforme. The exosomes successfully accumulated the drug at glioma locations (Rana et al., 2025).

Microbial-derived carriers, especially bacteria, are being explored as an innovative method to treat glioblastoma, a highly aggressive brain tumor, owing to their capacity to surmount diffusion barriers that impede drug delivery. These carriers can be designed to actively migrate towards and localize within tumors, potentially delivering therapeutic agents directly to malignant cells. The research demonstrates the development of a bacterium carrier, an avirulent strain of *Salmonella typhimurium*, proficient in active motility both in extracellular environments and within brain tumors. The bacterial carrier expresses p53, a tumor suppressor protein that facilitates apoptosis via both transcription-dependent and transcription-independent mechanisms. More than 50% of cancers with unfavorable clinical outcomes exhibit p53 mutations. Treatment strategies encompass reactivating mutated p53, reconstituting wild-type p53, or inhibiting MDM2; however, the absence of an effective delivery system for solid tumors has constrained the efficacy of these therapies. A bacterial vector expressing Azurin was engineered to augment apoptotic activity and stabilize wild-type p53. Azurin is a powerful tumoricidal protein that triggers apoptosis, obstructs receptor tyrosine kinase-mediated cell signaling, and inhibits angiogenesis. It stabilizes p53 by forming a complex that shields it from ubiquitination and proteasomal degradation. Azurin exhibits no cytotoxicity towards non-cancerous healthy cells and fails to permeate GBM cells or the blood-brain barrier. They manipulated p53 and Azurin expression in carriers through a hypoxic promoter pflE, effectively targeting the tumor microenvironment and inducing apoptosis in glioblastoma cells (Meh et al., 2017).

## 6 Mechanism of nanoparticles in counteracting chemotherapy resistance

In the last 10 years, nanoformulation has been investigated for its potential to reverse acquired resistance and reinstate drug efficacy in cancer treatment. Nanomedicines, such as pluronic, MDR modulators, and siRNA-based formulations, provide prolonged blood circulation and effective target accumulation, improving drug absorption via nanoparticle surfaces decorated with folic acid, peptides, and tumor receptor ligands (Wang et al., 2024). Metal nanoparticles enhance drug distribution, augmenting internalization within tumor cells, whereas TMZ alone results in merely 40% apoptotic cell death. Biomimetic nanosystems containing doxorubicin-encapsulated nanoparticles generate ROS.

**FIGURE 8:**
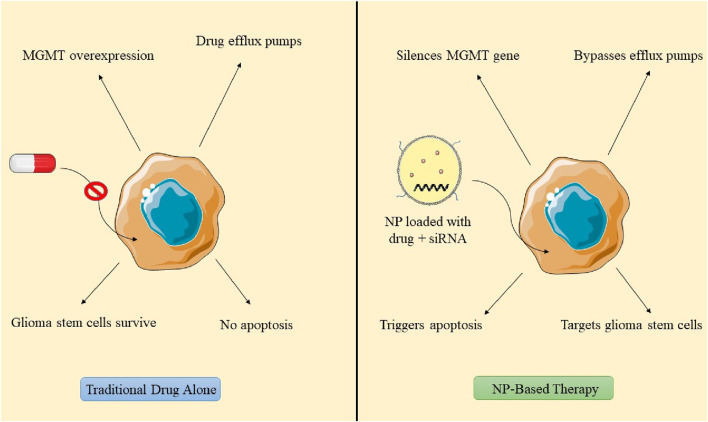
Mechanisms by which nanoparticles reverse chemoresistance in glioblastoma.

The reversal of chemoresistance entails the use of TMZ, traditional anticancer agents, or siRNA targeting the specific gene. Retinoic acid promotes the differentiation of GSCs into cancer cells, thereby enhancing apoptosis (Xu et al., 2022). Etoposide, which targets Topoisomerase II, may serve as an alternative treatment for TMZ-resistant GBM. NP-mediated gene therapy for reversing chemoresistance demonstrates favorable results when the design of nanoformulations and their structural organization effectively target tumor cells and facilitate endosomal escape. Conventional design models incorporate siRNA encapsulated within a nanoparticle adorned with outer surface ligands. Nonetheless, positive surface charges may result in membrane destabilization and heightened immune response. Researchers created PEI-siRNA polyplexes that enable endosomal escape following internalization, specifically targeting Gli1, a transcription factor in the Hedgehog signaling pathway. The PEIsiRNA polyplex effectively evades endolysosomal degradation via receptor binding and internalization, resulting in decreased expression of Gli1 and Smo, as well as a reduction in essential proliferative genes. This diminishes metabolic activity and the self-renewal of stem cells (Sevim et al., 2011; Di Filippo et al., 2021). Wang’s research employed magnetic carbon nanotubes conjugated with CD44 antibody for neoplasia therapy, demonstrating significant tumor reduction via endocytosis-mediated uptake and mitochondrial apoptosis in GBM cell lines G361 and G440 (Wang et al., 2023). NPs laden with cargo penetrates tumor periphery, internalizes cytoplasm, and engages with glioma stem cells. Stemness can be reversed via reactive oxygen species generation, RNA transcription inhibition, modification of the central dogma, and application of an external magnetic field, facilitating the differentiation of glioblastoma stem cells into normal drug-sensitive cells (Ertas et al., 2021; Figure 8).

## 7 Conclusion

GBM is a malignant astrocytoma characterized by a dismal prognosis and low survival rates, necessitating intricate treatment owing to its aggressive behavior, heterogeneity, and substantial obstacles posed by the blood-brain barrier and blood-tumor barrier. Nanotechnology presents a promising approach for glioblastoma multiforme patients by specifically targeting and eradicating GBM-initiating cells and cancer stem cells, thereby diminishing recurrence rates and treatment resistance. The EPR effect improves the solubility, stability, and bioavailability of chemotherapeutic agents by penetrating and maintaining polymeric, metallic, and lipid nanoparticles, including PLGA. Researchers altered lipid-based NPs to improve carrier specificity and uptake by GBM cells, reducing misdirected effects and adverse reactions, thereby offering a synergistic therapeutic approach.

## 8 Future outlook

Metallic nanoparticles improve imaging and photothermal therapy precision in tumor ablation, requiring comprehensive glioblastoma management via nanotechnology, emphasizing multifunctional nanoparticles for targeted treatment and real-time evaluation. NPs utilize CRISPR-Cas9 gene-editing to target glioblastoma mutations, facilitating precise delivery to tumor cells and imaging surveillance of the editing process and therapeutic results. This study explores the potential of nanotechnology in the treatment of GBM, emphasizing its promise while recognizing limitations related to research progress, clinical implementation, and inherent complexities. Our study emphasizes the significance of personalized therapies for GBM, underscoring the challenges related to design, implementation, safety, and accessibility. It underscores the potential of nanotechnology to surpass current therapies and enhance outcomes.
